# Drug Discovery and Repurposing for *Coccidioides*: A Systematic Review

**DOI:** 10.3390/jof11120875

**Published:** 2025-12-10

**Authors:** Sarah Saeger, Sofia Lozano, Nathan Wiederhold, Jieh-Juen Yu, Jose L. Lopez-Ribot, Chiung-Yu Hung

**Affiliations:** 1 Department of Molecular Microbiology and Immunology, South Texas Center for Emerging Infectious Diseases, The University of Texas at San Antonio, San Antonio, TX 78249, USA; ss7767@cumc.columbia.edu (S.S.); sofia.lozano@utsa.edu (S.L.); jose.lopezribot@utsa.edu (J.L.L.-R.); 2 Department of Pathology, The University of Texas at San Antonio, San Antonio, TX 78229, USA; wiederholdn@uthscsa.edu

**Keywords:** *Coccidioides*, coccidioidomycosis, valley fever, antifungal drugs, amphotericin B, fluconazole, drug resistance

## Abstract

*Coccidioides immitis* and *C. posadasii* are the causative agents of coccidioidomycosis (CM) or Valley Fever, endemic to the alkaline deserts of North and South America. Clinical treatment of CM is predominantly limited to the triazole and polyene drug classes. There are limited therapeutic options for the treatment of CM, most commonly requiring prolonged courses of therapy with established antifungal agents such as azoles and Amphotericin B, which often lead to toxicity and drug resistance. Clearly, there is a need to develop novel and better antifungal drugs against CM. This review examines both repurposed and recently discovered compounds in various stages of development for the treatment of CM.

## 1. Introduction

Coccidioidomycosis (CM), also known as Valley Fever, is an infection caused by the soil-borne fungi, *Coccidioides* species. CM has increasingly been recognized as a public health concern due to a current rise in cases and the climate-driven expansion of the endemic range of *Coccidioides* [1]. The etiologic agents of CM, *Coccidioides* species, are ascomycete fungi that grow in alkaline, sandy soils of the American semi-deserts. There are currently two recognized species, *C. immitis* (Ci) and *C. posadasii* (Cp). Ci is primarily endemic to California and Washington state. Cp is more geographically widespread and found in the arid regions of Arizona, Nevada, New Mexico, Utah, and Texas in the Southwest United States; Mexico, Guatemala, and Honduras in Central America; and Brazil, Argentina, Paraguay, Colombia, and Venezuela in South America [2,3]. Phenotypically and clinically, these two species are identical and can only be distinguished molecularly. Coccidioidomycosis is a nationally reportable disease in all of its endemic states except Texas [4,5]. The geographical range of *Coccidioides* is expected to increase due to changes in temperature and precipitation [6]. Recent reports indicate that trends in CM cases in California may be forecasted by examining patterns in temperature and precipitation. Specifically, dry years characterized by higher average temperatures are associated with increased CM cases [7], suggesting that rising temperatures could lead to a future surge in cases. CDC statistics show approximately 20,000 reported CM cases annually [8], though this number is likely an underestimation given the expanding endemic region and the lack of reporting in the endemic states of Texas. Case numbers in California tripled from 2014 to 2018, with an upward trend continuing each year [9]. Notably, CM accounts for 17% to 29% of community-acquired pneumonia cases in the Phoenix and Tucson metropolitan areas [8]. With the growing population of individuals aged 65 and older [10], the number of high-risk individuals is also increasing [8]. Most concerning, however, is that many states do not presently report their CM cases, or that many cases are misdiagnosed. The CDC estimates that the actual burden is 206,000 to 360,000 cases each year, a staggering 10 to 18 times the current reported numbers [8]. Altogether, those issues raise imminent concerns about treatment options against this growing threat.

As a dimorphic fungus, *Coccidioides* can exist in the environment as a mycelial mass consisting of filamentous hyphae segmenting into arthroconidia, which are released into the air and can be inhaled. Once they are inside a host, the arthroconidia transform into pathogenic spherules [11,12]. Spores can remain viable in the environment for extended periods and are dispersed by wind or soil disturbance. Within the lungs, arthroconidia undergo a dramatic restructuring, swelling isotropically into multinucleated rounded cells with a large central vacuole called spherule initials. As the spherule initials develop into spherules, they form internal septa, creating hundreds of separate compartments, which become individual endospores [11,13]. Within a few days, the mature spherule ruptures, releasing hundreds of endospores that continue to propagate local infection or, occasionally, disseminate to other organs. However, the mechanisms of extrapulmonary dissemination are poorly understood. In general, the maturation process takes five to seven days for a single spherule to produce 300-800 endospores in vitro. Despite the generation time in vivo being unknown, it is estimated to be much shorter, and within 1–2 days [11,13].

In most cases, the host’s immune response can contain the infection, and individuals remain asymptomatic (50–60%), but 30–40% experience mild, flu-like symptoms, including fever, cough, shortness of breath, chest pain, and headaches within 21 days of exposure [14]. These nonspecific symptoms are frequently misdiagnosed and referred to as community-acquired pneumonia [15]. About 5–10% of patients develop pulmonary sequelae, such as calcified nodules, granulomas, and lung cavitation, and 1–5% experience progressive dissemination from the lungs to the bones, joints, skin, and brain, which is often life-threatening if untreated [16]. Certain populations are at increased risk of severe or disseminated disease, including the immunocompromised (due to HIV/AIDs, cancer chemotherapy, diabetes, or organ transplantation), pregnant patients, and patients of African and Filipino ancestry [17]. Infections in these high-risk groups often require prolonged antifungal treatment and may result in substantial morbidity or mortality. Additionally, occupations involving soil disruption, such as farming, construction, or archaeology, may also increase exposure risk.

Fungal diagnostics have historically relied on serology, culture, direct microscopy, or histopathology. Still, these techniques are slow, low in sensitivity, and some require invasive biopsies, which delay treatment onset and dramatically impact patient outcomes [18,19,20]. Serologic testing remains the primary diagnostic method for confirming CM. According to the CDC [21] and the Infectious Diseases Society of America (IDSA) [16] guidelines, diagnosis and disease monitoring should begin with serologic assays, including enzyme immunoassays (EIAs) to detect IgG and IgM antibodies, often followed by immunodiffusion (ID) tests with tube precipitin (TP) and complement fixation (CF) antigen to confirm results. EIA tests offer rapid turnaround but have variable specificity (68–96%), and are therefore best used in combination with ID-CF and ID-TP [22]. In the case of a negative result but high suspicion or suggestive clinical signs, it is recommended to repeat the serology within 6 weeks, as antibodies may not be present at the time of initial symptom onset. Although it is not the primary diagnostic tool, direct visualization of *Coccidioides* spherules through microscopy or histopathology can provide supporting diagnostic evidence, particularly when spherules are identified in tissue or sputum samples [23]. Biopsy samples can be grown on agar plates for PCR diagnosis to confirm CM. The PCR method can also be employed for bronchoalveolar lavage and wash samples with good sensitivity, but it is less effective for other specimens [24,25,26]. Lateral flow assays (LFAs) can also detect IgG and IgM, but are less sensitive than EIA and are not considered suitable for disseminated samples or immunocompromised populations [22].

**Figure 1:**
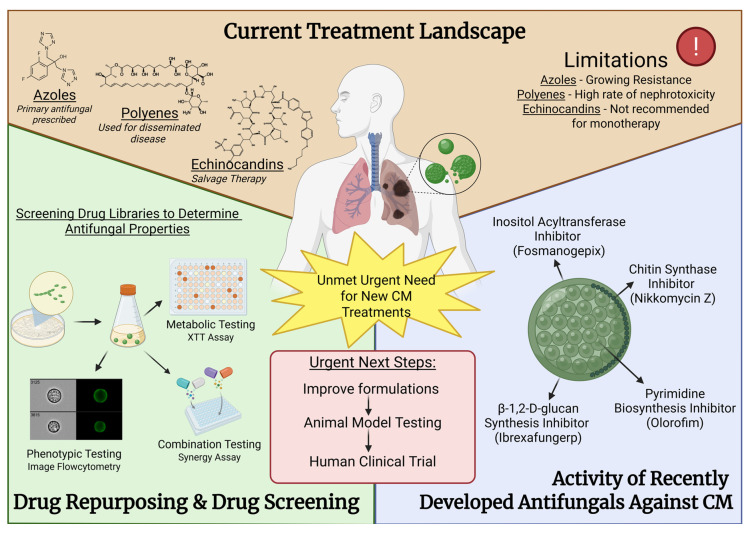
Current existing and novel anti-*Coccidioides* drugs, and summary of screening approaches to identify new compounds with antifungal activity against *Coccidioides* spp.

Current CM treatment options are limited to azoles such as fluconazole and itraconazole, and the polyenes amphotericin B [16,27,28]. The azoles, while effective in most contexts, are primarily fungistatic, often requiring prolonged or even lifelong therapy due to the risk of disease relapse. *Coccidioides* isolates with elevated minimum inhibitory concentration (MIC) values have also been observed for fluconazole [29]. Despite the growing burden of disease and the emergence of fluconazole-resistant isolates, there have been few advances in developing novel, targeted antifungals for *Coccidioides* or other fungal pathogens. This review highlights recent efforts to address this gap through drug repurposing, high-throughput screening, and surveys of emerging antifungal compounds undergoing clinical trials (Figure 1).

## 2. The Current Treatment Landscape

There are currently no antifungal drugs specifically approved for the treatment of coccidioidomycosis, so current disease management relies heavily on a limited set of agents primarily developed against other fungal species several decades ago. The first-line therapies use triazole antifungals, while amphotericin B is reserved for more severe, systemic infections. These azole agents, while effective in suppressing fungal growth, may not eradicate *Coccidioides*, especially in disseminated cases, but may improve host control of infection.

### 2.1. Azoles

The azoles are characterized by pentacyclic structures with either two or three nitrogen atoms, classified as either the imidazoles or triazoles, respectively. Due to their increased toxicity concerns, imidazoles like ketoconazole are no longer recommended for CM therapy and are limited to treating superficial mycoses. The preferred initial treatment for CM is the triazoles, including fluconazole (FLU), itraconazole (ITRA), posaconazole (POSA), voriconazole (VOR), and isavuconazole (ISAV). Triazoles inhibit the lanosterol 14-α-demethylase enzyme (Cyp51A or Erg11), which is required for the synthesis of ergosterol, a critical membrane sterol analogous to cholesterol [30].

FLU is the most widely used antifungal for CM due to its favorable pharmacokinetic and safety profile, excellent oral bioavailability, stable clearance (24–30 h half-life), and sound central nervous system (CNS) penetration [28]. It is commonly prescribed for both primary and chronic pulmonary infections and coccidioidal meningitis, except for pregnant patients due to potential risks of miscarriage and to the developing fetus [31]. Typical dosing ranges from 400 mg to 1200 mg daily, with higher doses (up to 2000 mg) used for CNS involvement [16,27,28]. However, the activity of FLU is fungistatic rather than fungicidal against *Coccidioides*, and long-term therapy is often required, sometimes indefinitely. Relapse after discontinuation of FLU can occur, especially in immunosuppressed patients. Additionally, recent work by Thompson et al. demonstrates that almost half of the *Coccidioides* isolates tested had elevated FLU minimum inhibitory concentrations (MICs) ≥ 16 µg/mL [29]. Although FLU is often the first-line antifungal for CM, the higher FLU MICs suggest that FLU may require higher doses to be effective. However, given the structural similarity and shared mechanisms of action of the azole compounds, it is possible that resistance could develop against azoles as a whole. Since CM is not a communicable disease, the source of this resistance may arise from an intrinsic resistance to FLU, the acquisition of de novo mutations under drug pressure in a host during treatment, or could even be driven by spore exposures to agricultural azole fungicides in endemic areas, which have been linked to azole and benzimidazole resistance in *A. fumigatus* [32,33]. However, the source of this resistance may be more convoluted, as *Coccidioides* spp. are not readily detected in agricultural soils of endemic areas [34]. Additional challenges presented by FLU for the long-term management of CM include adverse effects, particularly hepatotoxicity, abnormal heart rhythm, and interactions with other medications [35]. This increase in resistance and subsequent increase in effective dosage may also affect the overall cost of FLU treatments. For patients with chronic pneumonia due to CM, the 36-month cost of FLU therapy can range from approximately $47,000 to $55,000, depending on the dosage [36,37]. For disseminated infection, lifetime treatment for FLU may range from $315,000 to $321,000 [36,37]. These numbers solely cover the medication and do not account for lengthy hospital stays, loss of income, or any other charges associated with care.

ITRA is also used for the treatment of disseminated CM, particularly for non-meningeal disease, such as skeletal lesions [38]. It is generally considered to have greater in vitro potency against *Coccidioides* and may be effective in patients whose disease is unresolved by FLU [39,40]. However, ITRA’s use, especially the traditional encapsulated formulation, depends on adequate gastric acidity for dissolution. A newer formulation, SUBA-ITRA (super bioavailable ITRA), undergoes dissolution and absorption in the duodenum rather than relying on gastric acidity, improving bioavailability by 173% and reducing pharmacokinetic variability [41]. The spectrum of activity against *Candida* spp. is similar in ITRA and SUBA-ITRA formulations, but the enhanced bioavailability of SUBA-ITRA maintains consistent serum concentrations at lower doses than standard ITRA [42]. SUBA-ITRA is FDA-approved for antifungal prophylaxis in high-risk transplant patients and for the treatment of aspergillosis, histoplasmosis, and blastomycosis [43], but is still being clinically explored for CM [44]. A pilot trial (NCT03572049) demonstrated similar efficacy to conventional ITRA but fewer adverse reactions, and noted that concomitant proton-pump inhibitor use unexpectedly increased plasma concentrations [41]. A planned Phase II trial in CM patients intolerant or refractory to FLU (NCT04809649) was withdrawn due to COVID-1 related budget constraints, leaving its role in CM undefined. Like FLU, ITRA is also fungistatic, and the treatment course may last months to years, depending on disease severity and immune status. Therapeutic drug monitoring is often necessary to ensure adequate plasma concentrations and enhance clinical outcomes while avoiding toxicity.

VOR and POSA are second-generation triazoles structurally similar to FLU and ITRA, respectively. Both have been used off-label in refractory cases or in patients who cannot tolerate FLU or ITRA. POSA, in particular, shows strong in vitro activity against *Coccidioides* and good tissue penetration [45]. However, clinical data are limited, and these drugs are typically reserved for salvage therapy due to their cost and potential side effects, like hepatotoxicity and hormone disruption [46,47]. VOR is implicated in some additional adverse reactions, including visual disturbances, diffuse bone pain, and high photosensitivity, which increases the risk of squamous cell carcinoma [28,35]. VOR also requires frequent therapeutic drug monitoring due to non-linear pharmacokinetics and variable inter-patient and intra-patient bloodstream concentrations.

ISAV may offer some advantages in the treatment of CM due to its low in vitro MICs and favorable pharmacokinetic profile with reduced drug interactions and fewer adverse reactions compared to the other triazoles [48,49]. Although there is limited clinical data, existing studies show promising results indicating that ISAV may be useful in resolving or controlling both pulmonary and disseminated CM [50,51,52]. The prodrug of ISAV, isavuconazonium sulfate, has been approved for invasive aspergillosis and mucormycosis [53]. ISAV also shows promise in preclinical studies against *Coccidioides*, where it decreased CFU in a murine model of disseminated CM [54]. However, published clinical experience with the successful treatment of CM is limited, and its role remains undefined.

VT-1598 is a member of the tetrazole class of CYP51 inhibitors. The tetrazoles have the same mechanism of action as the triazoles, and have a broad spectrum of activity against *Coccidioides* [55], *Aspergillus* [56], *Cryptococcus* [57], and *Candida* [58,59]. To improve selectivity for fungal cells, the tetrazole group structurally replaces the metal-binding 1-(1,2,4-triazole) group, which reduces the CYP450-mediated drug–drug interactions of VOR, POSA, and ISAV [60]. Against both species of *Coccidioides*, VT-1598 demonstrated similar activity, with geometric mean MICs of 0.180 mg/L against *C. immitis* and 0.250 mg/L against *C. posadasii* [55]. In a murine model of CNS-disseminated CM, VT-1598 improved survival and reduced fungal burden [55]. A phase I clinical trial (NCT04208321) demonstrated that VT-1598 was well tolerated in healthy populations, and there were no serious adverse effects [60].

Oteseconazole (VT-1161) is another tetrazole being explored, which has a broad spectrum of activity against many species of *Candida* [61,62,63,64], *Rhizopus* [65], *Trichophyton* [66], and murine and canine CM [67,68]. VT-1161 has been approved for the treatment of recurrent vulvovaginal candidiasis (VVC) [69], and it is being investigated for onychomycosis, which has few non-topical treatments outside of terbinafine and ITRA, but is still in the early stages of investigation for invasive mycoses.

### 2.2. Polyenes

The macrolide polyene class of antifungals includes only two members: amphotericin B (AmB), which was approved by the FDA in 1959, and Nystatin. AmB is amphiphilic and acts by binding to ergosterol, potentially acting as either a sterol sponge to remove ergosterol from the cell membrane or by forming pores that allow for the efflux of K^+^ and Mg2^+^ and the influx of H^+^, acidifying the pH and killing the fungi [70,71]. Historically, the gold standard of treatment for severe and disseminated CM was AmB deoxycholate (AmBD) delivered parenterally. However, due to its high rate of nephrotoxicity, AmBD has largely been replaced by lipid formulations such as liposomal AmB (L-AmB) and AmB lipid complex (ABLC), which can reduce nephrotoxicity and infusion-related reactions [70,72,73,74,75]. AmB is often reserved for life-threatening infections such as azole-recalcitrant infections, fungemia, or disseminated diseases involving the bones, joints, or CNS. It is frequently used as an induction therapy before transitioning to oral triazoles for maintenance. Despite being fungicidal, the requirement of intravenous administration, monitoring for nephrotoxicity (azotemia) and electrolyte disturbances (hypokalemia and hypomagnesemia), and the high cost of the lipid formulations make it less suitable for long-term outpatient management.

### 2.3. Echinocandins

The echinocandin class of antifungals consists of four members: caspofungin (CAS), micafungin (MICA), anidulafungin (ANI), and the newest member, rezafungin (REZA). Echinocandins block the synthesis of β-1,3-D-glucan, which is an essential polysaccharide component of the fungal cell wall. However, echinocandins exhibit variable in vitro susceptibility to clinical isolates of *Coccidioides* [29,76,77,78] and only moderate in vivo efficacy during CM infection [79,80,81]. Therefore, they are mainly reserved for salvage therapy in azole-resistant infections and are not recommended for monotherapy.

### 2.4. Pyrimidines

Flucytosine, or 5-fluorocytosine (5-FC), is the sole member of the pyrimidine analog class of antifungals, which works by interfering with fungal DNA and RNA synthesis by replacing cytosine [82]. As resistance develops rapidly, 5-FC is not suitable for monotherapy and is often combined with AmB or azoles for the treatment of cryptococcal meningitis [83]. Unfortunately, this drug is ineffective against *Coccidioides,* even during combination therapy; thus, it is not recommended during the treatment of CM. Newer pyrimidine scaffold drugs with in vitro activity against molds, such as *Aspergillus*, are being investigated, and further preclinical development may eventually warrant combination testing against *Coccidioides* [84].

### 2.5. Unmet Needs

Despite these available agents, no antifungal drugs are currently FDA-approved specifically for the treatment of CM, and no new antifungal classes have been developed in decades. The prolonged time to symptom resolution, frequent relapses, and need for extended or lifelong therapy underscore the inadequacy of current treatment options. Furthermore, the limitations of these therapies, limited understanding of their fungistatic mechanisms, drug resistance, toxicity, and poor efficacy in the clinical presentations of CM, underscore the urgent need for novel antifungals with fungicidal activity. It is highly desired to identify novel classes of antifungals with improved pharmacokinetics that operate by novel mechanisms to avoid drug resistance. This need is particularly pressing in the context of the expanding geographic range of *Coccidioides* and growing disease burden in endemic regions, especially in immunocompromised populations.

## 3. Discovery and Development of Novel Chemotherapies for CM

Several approaches leveraging advances in new drug discovery and development are applied to address limitations of current CM therapy options, potential of drug resistance, toxicity, and inadequate tissue penetration.

### 3.1. Chitin Synthase Inhibitors

Nikkomycin Z (NikZ) is a secondary metabolite that has been found to possess potentially fungicidal properties [85,86]. As a chitin synthase inhibitor, NikZ has the potential to impact the structural integrity of various fungi, as chitin is a crucial component of fungal cell walls. These types of drugs are of special interest as their specific use against *Coccidioides* has been shown to impact the structural integrity, specifically of the spherules [87]. NikZ has demonstrated efficacy against *Coccidioides* in mouse models of pulmonary, central nervous system (CNS), and disseminated infections [88,89] and naturally infected dogs [90]. NikZ significantly increased the survival of mice infected with *Coccidioides immitis*, along with an average of MIC50 of 4 µg/mL (ranging from 1 to 16 µg/mL for various isolates) [86]. A Phase II trial showed that while the drug is safe for human use, the concentration in patients’ plasma decreases exponentially by the hour, proving to have a short lifespan in vivo [91]. The greatest challenge for the wider use of NikZ is to improve the chemical formulation to prolong the lifespan of this spherule-specific drug.

### 3.2. Olorofim

Olorofim (F901318) is a novel oral orotomide drug that inhibits dihydroorotate dehydrogenase (DHODH), a key enzyme in pyrimidine biosynthesis, which results in impaired fungal DNA synthesis [92]. This drug is highly specific to fungi and does not appear to affect analogous human enzymes at clinically relevant concentrations [93]. It has demonstrated fungicidal activity in murine models of CM [94]. Several Phase I clinical trials have established the pharmacodynamics, safety, and tolerability of this drug. Olorofim recently completed Phase IIb clinical trials on patients with refractory fungal infections (NCT03583164) [95]. Olorofim has moved into a Phase III trial to compare treatment outcomes with a liposome-formulated AmB (NCT05101187) against *Aspergillus* infection. The FDA has granted this drug a breakthrough therapy designation, and it is being investigated for the treatment of disseminated CM.

### 3.3. Ibrexafungerp

Ibrexafungerp (SCY-078) is an oral/IV triterpenoid antifungal that inhibits β-1,3-D-glucan synthesis, similar to echinocandins, ultimately disrupting fungal cell wall synthesis [96]. It is FDA approved for the treatment of vulvovaginal candidiasis (VVC) [97] but has not yet been explored in human CM cases. It has, however, demonstrated both in vitro [98] and in vivo [99] efficacy for CM. Further clinical trials are needed for treating CM.

### 3.4. Fosmanogepix

Fosmanogepix (APX001) is an oral/IV formulated first-in-class antifungal prodrug that converts to Manogepix (APX001A) in vivo. Manogepix inhibits the inositol acyltransferase Gwt1, which is essential for the attachment of cell wall proteins to glycosylphosphatidylinositol (GPI) anchors. It mainly impacts the biosynthesis of GPI-anchored mannoproteins, which compromise the integrity of the cell wall, disrupting fungal growth and adherence [100,101,102]. It has been tested for a broad-spectrum activity against several pathogenic fungi genera, *Candida*, *Cryptococcus*, *Fusarium*, *Aspergillus*, and *Rhizopus* [103], but not *Coccidioides* spp. A report showed that Manogepix has in vivo activity in a murine CM model [104]. It has received FDA Fast Track and Orphan Drug designations for several invasive fungal infections and it has begun Phase III clinical trials for candidemia and invasive candidiasis (NCT05421858).

## 4. Emerging Drug Discovery Strategies

Discovery of novel classes of antifungal drugs could be leveraged on a combination of modern molecular tools, improved screening methods, and new conceptual approaches, especially for *Coccidioides*.

### 4.1. Drug Repurposing and Drug Screening

Given the slow pace of new antifungal development and the unique challenges posed by *Coccidioides* species, drug repurposing may be one of the most practical approaches to expand treatment options for CM. Drug repurposing involves identifying new uses for existing compounds that have already been approved for other indications or have been extensively studied in humans [105]. This strategy offers several advantages: reduced development time and cost, established pharmacokinetics and safety profiles, and the potential to bypass many early-phase hurdles in novel drug development.

Unlike target-based screens, which require prior knowledge of essential fungal proteins or pathways, phenotypic screens enable the identification of active compounds with unknown, potentially novel mechanisms of action. Phenotypic screening tests compound libraries for their ability to alter fungal growth, morphology, or viability in vitro [106]. To date, only two reported studies have screened drug libraries for anti-*Coccidioides* activity, and this is likely due to the necessity of BSL3 precautions and because there is debate over the best screening morphology. We recently demonstrated that drug screening could be reliably recapitulated by the avirulent triple mutant (*C. posadasii* C735 ∆cts2/∆ard1/∆cts3; ΔT) of the parental *C. posadasii* C735 strain, which means that screening modalities would still be accessible to non-BSL3 laboratories, though hit confirmation in the virulent strain is still recommended [107]. The three mutations of the ΔT strain prevent the formation of mature spherules and endospores, limiting studies to arthroconidia and spherule initial development. Nonetheless, these early morphologic stages are central to the transition and progression of disease, preserving the model’s relevance. Furthermore, our group recently described the development and optimization of 96- and 384-well plate screening methodologies, enabling true high-throughput screening [108]. Recent screening efforts have employed either arthroconidia (environmental spores) or spherule initials (early parasitic form). No large-scale screening has been performed in more mature spherules or endospores, likely due to the heterogeneous nature of these cultures. Understanding the effects of morphology on drug efficacy may be important for *Coccidioides*, as transformations within the host could create conditions that make spherules more resistant to environmental stresses or immune clearance [13]. Image-based screening is an attractive method for identifying drug effects on spherules [109]. Screening platforms that incorporate two or more of these distinct forms may be more effective in predicting in vivo efficacy. The following section discusses compounds identified in library screenings, individual drugs tested against *Coccidioides* that were initially identified in screenings of other pathogenic fungi, and potential candidates warranting testing. It is important to note that the mechanism of antifungal activity observed may differ from the established mechanism of action of these drugs which have predominantly been described in mammalian models.

### 4.2. Anti-Infective Agents

Niclosamide (NIC) and its ethanolamine salt (NEN), along with rafoxanide (RAF), oxyclozanide (OXY), closantel (CLO) and its sodium salt (CLN), and resorantel (RES) have demonstrated antifungal activity against 24 h spherule initials of *C. posadasii* C735 and the spherule initials of *C. posadasii* ΔT in a 10 µM screening of the Selleck Antiparasitics L8200 library [107]. NIC and NEN were top performers in the screening with low MIC50s (0.24 µg/mL and 0.21 µg/mL, respectively) and demonstrated synergistic activity when combined with AmB. NIC also demonstrated additional activity against *C. posadasii* ΔT arthroconidia at 1 µM and 5 µM by Mead et al. [110]. These compounds from the halogenated salicylanilide drug class, traditionally used as anthelmintic antiparasitics, are thought to disrupt mitochondrial function and oxidative phosphorylation [107,111,112]. Interestingly, halogenated salicylanilides have emerged as hits in screens of other fungi, including NIC in *Candida albicans* and *Candida auris*, *Histoplasma capsulatum*, *Paracoccidioides brasiliensis*, *Sporothrix brasiliensis*, and *Cryptococcus neoformans* [109]. OXY has shown activity against *C. albicans*, *Paracoccidioides* and *Histoplasma* [113]. RAF inhibits the growth of *C. albicans* and *Aspergillus fumigatus*, and CLO was effective against *Trichophyton tonsurans* [114]. NIC’s repurposing potential is limited by its recommendation only to be administered orally or by nebulization, as it has adverse effects when administered intravenously [111,115]. Nonetheless, NIC derivatives and an appropriate formulation may be further investigated in vivo against CM. Two additional antiparasitics outside of this class also showed inhibitory effects against 24 h spherule initials in vitro: Dichlorophene (DICH), a veterinary anthelmintic for tapeworms, and triclabendazole (TRIC), used for treating fascioliasis, have also been identified in drug screenings against *C. auris* and *C. neoformans.* Interestingly, DICH did not show activity in the comparative screening with *C. posadasii* ΔT, which requires follow-up but may indicate that the mutant lacks pathway(s) essential to DICH’s activity in *Coccidioides*.

Pentamidine isethionate (PENT-I) is initially administered intramuscularly for the treatment of protozoal infections, including trypanosomiasis and cutaneous and visceral leishmaniasis. PENT-I also has efficacy against *Pneumocystis pneumonia* (PCP) [116] and is administered as an aerosol to prevent or treat this fungal infection [117]. PENT-I has demonstrated antifungal activity in vitro in *C. auris* [118], *C. neoformans* [118], *Fusarium oxysporum* [119], and *Coccidioides* [107]. It acts by disrupting mitochondrial membrane potential and DNA/RNA synthesis [118], but it can also act as an anti-inflammatory agent or an antagonist of the calmodulin, histone acetyltransferase, and NMDA receptor [116,120,121]. Despite its broad in vitro activity, toxicity, and narrow therapeutic windows, its repurposing for the treatment of systemic fungal infections has been limited.

### 4.3. Antineoplastic Agents

Antineoplastics may disrupt cell signaling, cell cycle regulation, and membrane integrity. However, there is a substantial risk of off-target effects on human cells, given that many are already used in cancer treatment. Two tyrosine kinase inhibitors were found in vitro drug screenings: tyrphostin-A9 (TYR9), a platelet-derived growth factor receptor (PDGFR) inhibitor, and tyrphostin-AG-879 (TYR), an ErbB2 inhibitor [122,123,124,125]. The spherule screening found two additional tyrosine kinases: SU014813 (SUN) is a multi-receptor PDGFR/VEGFR2 inhibitor [126], while DCC-2618 (RIP) is a switch controller for PDGFR [127]. Notably, 10058-F4, a c-myc inhibitor, was one of the top compounds against spherule initials with an MIC50 of 0.78 µg/mL and moderate synergy with AmB, and has previously been identified in drug screenings for *C. neoformans* [128]. Tamoxifen is a selective estrogen receptor modulator from the arthroconidia screening, similar to toremifene (TOR) from the spherule screening. These drugs have been used to manage metastatic breast cancer [129]. In vivo tamoxifen combined with FLU reduced fungal burden to near zero and prevented dissemination to the spleen by day 8 compared to the FLU alone control in a murine model of pulmonary CM [110]. The promising in vivo data for tamoxifen suggest it is a top candidate for further investigation. Although based on a clinical trial in patients with cryptococcal meningitis, the doses may need to be adjusted to achieve fungicidal activity [130]. Given the structural similarity of TOR to tamoxifen, TOR warrants further testing as a CM treatment in animal models, as it could overcome the limitations encountered in the tamoxifen clinical trial.

### 4.4. Anti-Inflammatory Agents

This group includes five compounds of interest. SKF-86002 (SKF), a p38 MAPK inhibitor [131]; triamcinolone (TRIO), a corticosteroid [132]; BAY-11-7082 (BAY), an NF-κB inhibitor [133]; and bortezomib (BZB), a 26S proteasome inhibitor and anticancer drug [134,135]. Their antifungal effects may stem from a modulation of stress response pathways and inhibition of protein degradation systems. SKF may play a similar role to another potential antifungal, SQ109, which induces an anti-microbial M1 macrophage polarization and a protective Th1 and Th17 response [136] through activation of the p38 MAPK pathway [137]. TRIO may present several challenges for repurposing due to its poor water solubility and significant adverse effects, including adrenal suppression, osteoporosis, cataracts, and growth stunting in children [132]. In the arthroconidia screening, Mead et al. also discovered BAY-11-7085 [110], another NF-κB inhibitor similar to BAY-11-7082, which also inhibits IκBα phosphorylation, a cellular signaling of inflammasome activation in humans, and a trigger for apoptosis [138,139]. Both BAY agents have also been identified in a screening against *C. albicans* biofilms [140]. However, BAY-11-7082 was ineffective against a murine model of disseminated candidiasis [141]. Since fungi lack an inflammasome, the mechanism of action of these drugs is unknown.

### 4.5. Neurologic Agents

LY310762 (LYN) is a selective antagonist of the 5-hydroxytryptamine 1D (5-HT1D) serotonin receptor, VU-0422288 (VUN) is a glutamatergic modulator targeting the metabotropic glutamate seven receptor (mGluR7), and ST-1859 (STN) is an anti-amyloidogenic compound that inhibits Aβ aggregation [142]. While these neurologic targets are not conserved in fungi, their antifungal activity may be useful for treating CNS dissemination. Sertraline, a selective serotonin reuptake inhibitor (SSRI), provides further support for neurologically active drugs as antifungal candidates. Mead et al. reported that sertraline exhibited fungicidal activity against *Coccidioides* arthroconidia in vitro, a finding subsequently recapitulated in vitro in *C. immitis* by Paul et al. [110,143]. In vivo, sertraline modestly prolonged survival in a murine CM model when used as monotherapy, although its efficacy was insufficient to support its clinical use as a standalone agent [110]. Sertraline has also demonstrated in vitro and in vivo antifungal activity against *C. neoformans* that was synergistic with FLU, and is thought to have inhibited translation and disrupted lipid metabolism, evidenced by the production of supersized lipid droplets [144]. Despite promising data demonstrating CSF clearance, advancement has been limited due to not reducing mortality nor increasing the rate of fungal clearance in a Phase III clinical trial (NCT01802385) of HIV-associated cryptococcal meningitis [145]. Additionally, serotonergic agents may interact with host neurotransmitter systems, presenting safety concerns for chronic use.

### 4.6. Analgesics and Miscellaneous

PD-198306 (PDN), a MAPK/ERK kinase inhibitor, and bardoxolone (BOX), an antioxidant inflammation modulator that inhibits nitric oxide synthase, have been observed to inhibit spherule growth, possibly through interference with the aforementioned signaling pathways and oxidative stress responses.

Meglumine (MEG), depending on its formulation, is a hexosamine that can be useful as an excipient to prepare a diagnostic contrast medium or as an antiprotozoal to treat leishmaniasis [146]. Its mechanism of action warrants further investigation.

Auranofin (AUR) is a gold-containing anti-rheumatic drug that has demonstrated broad-spectrum antifungal activity in vitro, including against *Candida* [147,148], *Histoplasma* [149], *Cryptococcus* [150,151], *Aspergillus* [148,152], *Scedosporium* spp. [148,153], and *Coccidioides* [107]. Its primary mechanism involves the inhibition of fungal thioredoxin reductase, leading to disruption of redox homeostasis and the accumulation of reactive oxygen species [154]. However, progress in the preclinical development of AUR has been limited, potentially due to concerns about host toxicity, off-target effects, and the more favorable pharmacokinetics of newer antifungal agents. Since disseminated CM can cause desert rheumatism, this drug could have the potential to treat the causative agents and the symptoms simultaneously.

Oxethazaine (OXE, oxetacaine), a potent topical anesthetic, has recently been identified as a promising antifungal compound. It exhibits fungicidal activity at micromolar ranges against *Cryptococcus* [109] and *Coccidioides* [107]. Its exact mechanism of action remains under investigation, but membrane disruption and inhibition of proton pumps have been proposed [155]. Further pharmacologic characterization work in animal models is necessary to determine its feasibility for systemic CM treatment.

## 5. In Vivo Models and Translational Tools

Robust in vivo models are critical for bridging the gap between in vitro screening and clinical application in *Coccidioides* drug discovery. Improved CM models facilitate the evaluation of therapeutic efficacy, tolerability, and pharmacokinetics, using readouts such as survival, weight loss, and fungal burden quantified by colony-forming units (CFUs), histopathology, or molecular tools such as qPCR. Different preclinical models, including the *Galleria mellonella* larvae and murine models of pulmonary and disseminated CM, as summarized in Table 1, offer complementary strengths for all stages of antifungal testing.

The *Galleria mellonella* (wax moth larvae) infection model has gained traction as a rapid, low-cost, and flexible system for initial antifungal screening [156,157]. Larvae can be infected with *Coccidioides* arthroconidia by injecting the spores into the haemocoel via the proleg and grow at temperatures above 37 °C [158]. Subsequently, drug delivery is straightforward: the larvae can be treated with candidate drugs by injection into the opposite proleg, and their survival tracked over several days, followed by a CFU readout or histology to compare fungal clearance [158]. Since *Coccidioides* can complete its whole life cycle within the larvae, this model has several advantages: the larvae can be maintained without special housing, needing only Petri dishes, and their silk is removed daily to prevent them from becoming moths [158]. *Galleria* lacks adaptive immunity but does possess innate immune pathways (e.g., Toll-like receptors and melanization cascades) that mimic early antifungal defenses in mammals [159,160]. The model’s ease of use enables extensive, high-content studies to test multiple drug candidates or different doses simultaneously in a cost-effective manner [158,161,162,163]. Despite these benefits, the *Galleria* model has limitations [164]. It represents an infection more akin to cutaneous CM than to pulmonary CM, cannot recapitulate mammalian pharmacokinetics because it lacks key organs involved in drug distribution, metabolism, and excretion, and cannot capture CNS or disseminated disease. Therefore, while *Galleria* is useful for early triage and prioritization of hits from in vitro screens, findings require validation in mammalian systems.

Although experimental models exist for rats, guinea pigs, dogs, and non-human primates, mouse models remain the gold standard for preclinical evaluation of anti-*Coccidioides* drugs due to their ability to recapitulate key aspects of human disease, including pulmonary infection, CNS dissemination, and immune-driven granulomatous infection [165]. Mice can be infected via the oropharyngeal or intranasal route with arthroconidia to establish primary lung infections, which will then disseminate lethally to the spleen, brain, and other organs within 2 to 4 weeks post-infection with a virulent isolate of *Coccidioides* [166,167,168]. Infection and treatment efficacy can be assessed via survival and fungal burden or tissue pathology of the lungs, spleen, and brain. Due to the nature of the model, it is recommended that routine pharmacokinetic and pharmacodynamic testing be performed separately from drug efficacy testing in infected mice, unless the equipment is readily available within the BSL-3 facility. The key strengths of the murine model are the ability to survey the mouse immune system and assess host-pathogen interactions and immune modulation by antifungal agents [169]. In vivo testing is especially important given the frequent disconnect between in vitro MICs and in vivo efficacy [169,170,171]. Promising drugs may fail due to poor bioavailability, metabolic instability, immunomodulatory effects, or inability to reach infected tissues at effective concentrations. Conversely, some agents with modest in vitro activity can exhibit substantial therapeutic benefits in vivo, highlighting the value of whole-organism studies.

## 6. Challenges and Opportunities

One of the most pressing challenges in antifungal development for *Coccidioides* is the lack of standardized antifungal susceptibility testing (AFST) protocols tailored to its unique biology. The Clinical and Laboratory Standards Institute (CLSI) provides general guidance for testing against dimorphic fungi in the M38 standard, but interpretive breakpoints specific to *Coccidioides* have not been established, complicating comparisons across studies and hindering clinical decision-making [172]. Compounding this, *Coccidioides* transitions from arthroconidia into host-specific forms, spherule initials, mature spherules, and endospores, which may vary in susceptibility to antifungal agents. Since current AFST testing relies on in vitro testing of arthroconidia, stage-specific activity relevant to host infection may be missed [173]. Another significant barrier is assessing drug penetration into granulomas, which serve as physical and immunological barriers in chronic CM, and a source of relapse [174]. Granulomatous structures are often hypoxic, immunologically complex, and poorly perfused, raising questions about whether antifungal agents can effectively reach and act within these sites [174]. High spatial resolution Mass spectrometry imaging can elucidate drug penetration into granulomas, but no therapies have been optimized specifically for granuloma penetration or targeting. There are also no in vitro organoid-like granuloma models for CM, though they do exist for tuberculosis and could theoretically be repurposed [175]. Clinical trial recruitment poses additional hurdles to therapeutic testing and development. Due to the endemic nature of CM, clinical trial enrollment is geographically restrained, with eligible participants often dispersed across broader areas [2,3]. This makes multicenter trials difficult and slows the pace of therapeutic evaluation. Finally, there is a paucity of in vivo data for many compounds identified through repurposing or high-throughput screenings. Without robust animal efficacy studies, it remains challenging to predict which drugs have translational potential beyond cell culture analysis.

Despite these challenges, several promising opportunities exist to accelerate antifungal drug discovery for *Coccidioides*. First, using the ΔT strain for preliminary drug screenings would greatly expand access for any BSL-2 laboratory equipped with appropriate biosafety cabinets. And so long as they had the equipment, labs could screen both arthroconidia and spherule initials, as the mutant does not produce more mature spherules or endospores [168]. Next, drug screenings across different or multiple *Coccidioides* cell types would provide a refined strategy to identify compounds targeting the parasitic forms of *Coccidioides*, which are most relevant to host infection. The screening approach for spherules has already yielded promising hits that may have been missed in arthroconidia screens [107]. Combination screening is another underexplored or promising area. Pairing existing antifungals or adjuvants, such as efflux pump inhibitors, with screening libraries may yield compounds that enhance efficacy, overcome resistance, and reduce the required dose of each agent compared to drug-alone controls. Together, these opportunities highlight a path forward for more effective and targeted antifungal therapies for CM. These advancements have the potential to expand the therapeutic arsenal against this challenging infection.

## Figures and Tables

**Table 1:** Experimental models for CM drug testing.

Model	System Type	Use in CM Research	Strengths	Limitations
In vitro MICs	Cell-based	Primary screen	High-throughput, stage-specific assays	Poor predictor of in vivo efficacy
*Galleria mellonella*	Invertebrate	Initial in vivo model	Cost-effective, dose–response model	Lacks organs associated with ADME *
Murine (lung)	Mammalian	Pulmonary model	Mimics primary CM site	Ethical cost, variable strain virulence
Murine (disseminated)	Mammalian	CNS/Spleen dissemination	Assesses systemic spread	Requires complex endpoints
Murine (CNS)	Mammalian	Treatment of CNS infection	Specifically assesses drug efficacy in CNS infections	Requires expertise in CNS inoculation

* ADME: absorption, distribution, metabolism, and excretion.

## Data Availability

No new data were created or analyzed in this study. Data sharing is not applicable to this article.
